# Evaluation of adherence by elderly nursing home patients to regular consumption of apple compote enriched with protein and soluble fiber

**DOI:** 10.1007/s40520-015-0415-3

**Published:** 2015-08-04

**Authors:** F. A. Allaert, L. Guérin-Deremaux, A. Mauray-Soulier, M. H. Saniez-Degrave

**Affiliations:** CEN Nutriment, impasse Françoise Dolto, 21000 Dijon, France; Roquette, 62080 Lestrem, France

**Keywords:** Protein, Fiber, Sarcopenia, Adherence, Elderly

## Abstract

**Background:**

An increase in daily doses of protein and fiber for the elderly is relevant in preventing sarcopenia and preserving intestinal balance. However, such intake of supplements is often compromised by the lack of adherence among the elderly.

**Objectives:**

The main objective was to evaluate the perception of the hedonic qualities of compote enriched with NUTRALYS^®^ pea protein, NUTRALYS^®^W hydrolyzed wheat gluten and NUTRIOSE^®^ soluble fiber and the changes in that perception due to repeated consumption. The secondary objectives were to evaluate the evolution in the quantity of compote eaten, satisfaction with consumption and any changes in fatigue, digestive comfort and digestive tolerance when eating compote every other day for 3 weeks.

**Method:**

An observational study was conducted in nursing homes on volunteers aged 70–90 years. The compote was proposed as a lunchtime dessert every two days for a period of three consecutive weeks. All criteria were evaluated at days D0 and/or D1, D7, D15 and D21, except for the amount of compote eaten, evaluated after each meal at which it was served.

**Results:**

When first tasted, the compote was judged ‘rather pleasant’ to ‘very pleasant’ by 91.6 % and this rating held up at 79.2 % (*p* = 0.1797) after 1 week, 83.3 % (*p* = 0.3173) after 2 weeks and 79.2 % (*p* = 0.2568) after 3 weeks. Average consumption of compote was stable and varied between a maximum of 79.5 % of the total quantity at inclusion to a minimum of 61.5 % recorded on D17. The other parameters did not change significantly.

**Conclusion:**

Pea protein, hydrolyzed wheat gluten and soluble fiber seem to provide an appropriate form of protein and fiber supplementation in the diets of elderly people in nursing homes.

## Introduction

Longer life expectancy means that one of the major public health challenges for the coming years will be keeping people independent for as long as possible. Independence involves maintaining muscle capital, but whereas much effort has been put into preventing osteoporosis muscle function remains neglected even though it is just as essential.

Many studies have shown that from the age of 35 years, muscle structures tend to deteriorate spontaneously. This phenomenon increases with age due to various factors, including the lack of physical activity and reduced protein intake [1–3]. In the INCA 2 study of men aged 55–79 years, an 8 % average reduction in protein consumption was observed compared with 18- to 34-year-old men [4].

Among younger senior citizens of both sexes between the ages of 65 and 75 years, the first effects of this deterioration are felt, manifesting itself as a loss of muscle strength which is mostly felt as ‘muscle fatigue’. Eventually, it entails an increased risk of falls, increasing incapacity to perform habitual daily movements and loss of independence, leading to increased morbidity and mortality [5, 6]. Just as calcium and vitamin D are needed to support bone metabolism, muscle metabolism also requires essential amino acids for protein synthesis.

Ensuring the protein ration of the elderly is an important concern in preventing sarcopenia [7]. The recommended nutritional intake of proteins for the elderly is at least 0.8 g kg^−1^ d^−1^ [4]. Some researchers even recommend an intake of 25–30 g of high-quality protein per meal [8].

Because elderly people often have difficulty chewing and lose their appetite for meat, an oral nutritional supplement is commonly served in nursing homes to ensure the optimum level of protein intake. However, intake of such supplements is often compromised by residents’ lack of adherence: most products are abandoned after some weeks because of the quantities required to be eaten, or by their taste or texture. In this context, vegetable proteins might have two advantages: they are a quality source of protein because they are easily digested; and they can be incorporated into various foodstuffs to diversify the diet and elicit gustative interest. To test this hypothesis and the hedonic qualities in particular, an apple compote with biscuit flavoring and enriched with pea protein, hydrolyzed wheat gluten and soluble fiber was served.

## Method

### Study objectives

The main objective of this observational study was to evaluate elderly persons’ perception of the hedonic qualities of this protein- and fiber-enriched compote in the nursing home setting and any change in their perception over the course of repeated consumption for 3 weeks. The secondary objectives relate to the evaluation of the quantity of compote eaten by the volunteers, their level of satisfaction and changes in their fatigue levels and digestive comfort and tolerance.

### Evaluation criteria

The principal study criterion was the definition of the compote’s hedonic quality on a six-point Likert scale varying from ‘very pleasant’ to ‘very unpleasant’ [9] measured on the first day of consumption D1, followed by changes therein after 7 days (D7), 15 days (D15) and 21 days (D21) of regular consumption every other day as a lunchtime dessert. The secondary criteria pertaining to changes are:

- consumption of the compote evaluated by weighing the quantity remaining after lunch;
- satisfaction with the time of eating and the quantity of compote served as well as satisfaction with taste, texture or appearance of the compote using verbal questionnaires;
- fatigue evaluated by the Pichot scale [10];
- digestive comfort evaluated by the first eight questions on the Gastrointestinal Quality of Life Index as adapted [11];
- and evaluation of the digestive tolerance by a simple declaration by the volunteers in the event of digestive disorders.

### Volunteer selection criteria

In the context of an observational study, it was important not to influence in any way the behavior of the carers and meal-time staff and their relations with the residents. To reflect the general population of people in nursing homes, the selection criteria were voluntarily limited to men and women aged 70–90 years who liked the compote and biscuit flavor and who were intellectually capable of understanding the observational study they were invited to participate in as well as of replying to a series of questions about their dietary preferences.

So as not to distort the observational conditions, no non-inclusion criteria were required of the elderly apart from their not meeting the inclusion criteria or declining to take part in the study; not being allergic to the constituents of the product under study; being already enrolled in another observational study or therapeutic trial; and having a degree of dependency below GIR 3 on the AGGIR (Autonomie Gérontologie Groupes Iso-Ressources) rating chart [12].

### Description of volunteers

24 volunteers were recruited in two nursing homes in the Dijon area (France). Two volunteers with a GIR dependency score less than 3 were maintained in the study as they were deemed capable of participating in the evaluation. Three volunteers were removed during the study because of a sporadic digestive disorder which they ascribed to eating the compote, and thus the rules set out in “Data analysis” were applied to the data for those subjects.

The 24 volunteers maintained in the analysis were aged on average 83.7 ± 6.2 years (min 70.0, max 93.0) and 62.5 % of them were women. Of the volunteers, 25.0 % were overweight and 20.8 % obese. Two (8.3 %) had an AGGIR score of 2, 12 (50.0 %) of 3, 7 (29.2 %) of 4 and 3 (12.5 %) of 5. Their fatigue evaluated on the Pichot scale ranging from 0 (no fatigue) to 32 (extreme fatigue) was 12.9 ± 8.5 on a scale. Six of them (25.0 %) felt excessive fatigue defined on the Pichot scale by a score of more than 19. Their digestive comfort evaluated by the Gastrointestinal Quality of Life Index was 27.8 ± 3.5 for an optimal maximum of 32 (Table 1).

**Table 1:** Evolution of evaluation criteria

Criteria	D0	D1	D7	D15	D21	*p* value
Hedonic satisfaction	NA	4.6 ± 0.9	4.2 ± 1.3	4.2 ± 1.0	4.1 ± 1.2	0.3190
Fatigue score (Pichot)	12.9 ± 8.5	NA	14.2 ± 9.4	12.3 ± 8.0	12.8 ± 8.2	0.4199
Digestive comfort score	27.8 ± 3.5	29.9 ± 2.3	28.1 ± 4.0	27.2 ± 4.5	28.1 ± 3.4	0.0134

Values are expressed as mean ± standard deviation

*NA* not applicable

### Product composition

The compote composition was as follows: stewed apples (55.0 %), water (32.4 %), NUTRIOSE^®^ soluble fiber (6.3 %), NUTRALYS^®^ XF pea protein (5.1 %), NUTRALYS^®^W hydrolyzed wheat gluten (0.9 %) and biscuit flavoring (0.3 %). The NUTRALYS^®^ proteins and NUTRIOSE^®^ fiber were supplied by ROQUETTE (Lestrem, France). The nutrition level of the product is presented in Table 2. A serving of 125 g of apple compote was offered to the volunteers. The serving provided 6.7 g of fiber and a total 6.4 g of protein. So as not to induce bias into the evaluation of the change in dietary intake of compote and to abide by the ‘naturally occurring’ conditions of an observational study, the meal-time staff were not given any specific instructions. If the product was rejected by a volunteer, the staff then proposed an alternative dessert in the usual way.

**Table 2:** Nutritional values for 100 g

Calories (kcal)	101.0
Protein (g)	5.0
Carbohydrate (g)	16.6
Fat (g)	0.4
Dietary fiber (g)	6.0

### Conduct of the study

The study lasted 3 weeks for each volunteer. The compote under study was proposed as a lunchtime dessert every two days. To evaluate the change in consumption of compote during the study, the staff recovered the compote tubs after each meal (even empty) and weighed them. The hedonic quality of the compote, the satisfaction obtained from eating the compote and the evaluation of digestive tolerance were noted on observation cards at D1, and then at the end of each week (D7, D15, D21). Fatigue and digestive comfort were assessed at inclusion and then on D7, D15 and D21.

### Data analysis

The analysis was performed under ‘everyday conditions’. It related to the volunteers who ate and rated the compote upon inclusion whatever the subsequent changes and whatever their adherence to the protocol, the latter reflecting behavior closest to real life. To incorporate the evaluation of behavior of volunteers who dropped out of the study, the following rules were applied:

- For taste rating, fatigue scale and digestive comfort, the LOCF (last observation carried forward) method was used by attributing the final known value from when a volunteer dropped out of the study, through to study completion (as initially planned by the protocol).
- For the weighing of the compote after meals, consumption was taken to be zero from the time a volunteer dropped out of the study, through to its completion as initially planned by the protocol.

Qualitative variables are described by frequencies and percentage; quantitative variables by means and standard deviations. Percentage comparisons over the course of the various follow-up visits were made using McNemar tests for paired variables; by Wilcoxon rank tests on repeated series for multimodal qualitative variables; and by analysis of variance on repeated series for quantitative variables. The data were recorded on Capture system software and the statistical analyses conducted using SAS version 9.2 software.

## Results

### Evolution of hedonic satisfaction

**Fig. 1:**
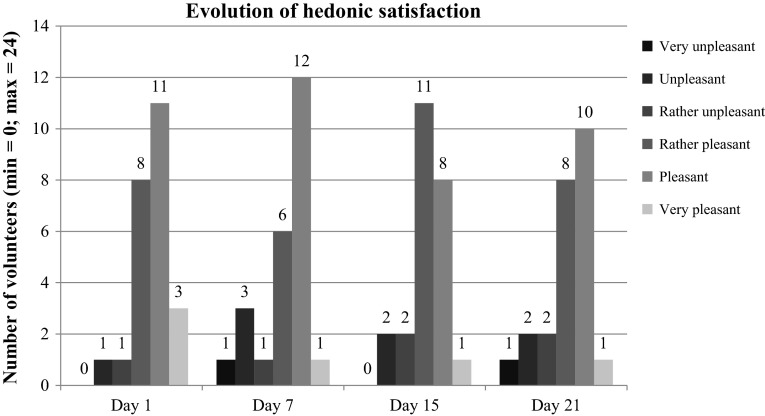
Evolution of hedonic satisfaction

On D1 after eating the first compote, it was found to be ‘rather pleasant’ to ‘very pleasant’ by 22 out of 24 volunteers, or 91.6 % (14 of whom rated it ‘pleasant’ and ‘very pleasant’, i.e., 58.3 %) (Fig. 1). The mean value of hedonic satisfaction was 4.6 ± 0.9 out of a maximum of 6.

Perception of the taste of the compote did not vary statistically over the 3 weeks: 19 volunteers finding it ‘rather pleasant’ to ‘very pleasant’ (79.2 %) after 1 week (*p* = 0.1797); 20 volunteers (83.3 %) finding the same after 2 weeks (*p* = 0.3173); and finally 19 volunteers (79.2 %) likewise after 3 weeks (*p* = 0.2568).

The main variation occurred over the first week with a decline in hedonic satisfaction of 12.5 %. This was essentially a slide in opinions from ‘very pleasant’ to ‘pleasant’, after which appreciation ratings remained very stable.

In terms of average hedonic satisfaction, opinions also remained stable, varying from 4.6 ± 0.9 on inclusion to 4.1 ± 1.2 after 3 weeks (*p* = 0.3190) (Table 1). Volunteers who did not like the taste judged it not sweet enough or else too runny or too lumpy. None of these dislikes of taste put an end to the study.

### Quantity of compote consumed

**Fig. 2:**
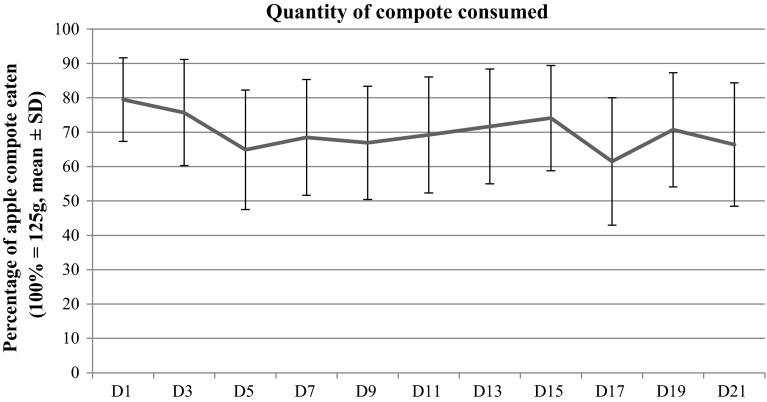
Compote consumption variation

Mean consumption of compote was stable throughout the 3 weeks of the study and varied between a maximum upon inclusion of 79.5 % of the total quantity (99 g) and a minimum of 61.5 % (77 g) recorded on D17 (Fig. 2). When expressed in terms of the median, the intake of compote appears even more stable, with one volunteer out of two consuming at least 85 % (106 g) of the total quantity of compote (125 g) throughout the study.

### Perception of times of eating and quantity served

Eating the compote at lunchtime was enjoyed by 17 out of 24 volunteers (70.8 %) upon inclusion. The percentage remained stable among those volunteers, who remained in the study until its completion: 73.9 % on D7, 77.3 % on D15 and 71.4 % on D21.

Among the volunteers who found this was not the best time, the alternative shifted over the weeks more often toward taking it at afternoon teatime rather than evening dinnertime. Four out of seven volunteers on D1, four out of six volunteers on D7, four out of five on D15 and five out of six on D21 preferred taking the compote at afternoon tea time. As for appreciations of the quantity served, it was judged sufficient by more than three-quarters of the volunteers throughout the study; for the others, the quantity was mostly thought excessive.

### Fatigue score

**Fig. 3:**
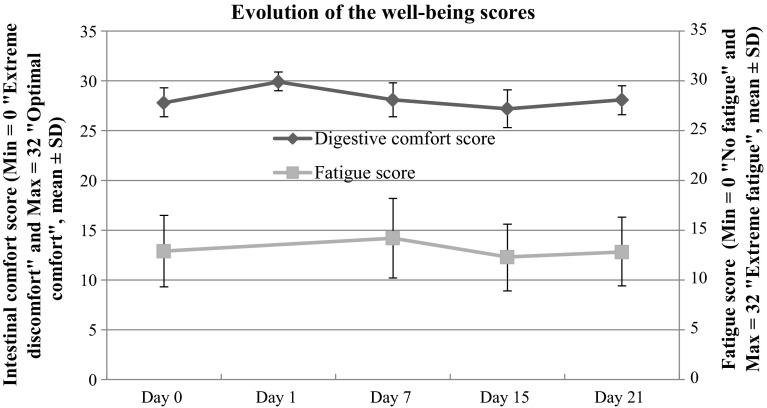
Evolution of the well-being scores

Fatigue evaluated on the Pichot scale was stable, varying from 12.9 ± 8.5 on a scale ranging from 0 (no fatigue) to 32 (extreme fatigue) upon inclusion to 12.8 ± 8.2 after 3 weeks (Fig. 3); (Table 1). The results did not change significantly over the course of the study.

### Digestive comfort score

The digestive comfort of subjects was stable throughout the 3 weeks of the study with non-significant variations between inclusion and the end of the study (Fig. 3); (Table 1). The score varied from 27.7 ± 3.5 upon inclusion on a scale ranging from 0 (extremely uncomfortable) to 32 (optimal comfort) to a score of 28.1 ± 3.4 after 3 weeks.

### Digestive tolerance

Twenty-one of the 24 volunteers included in the study did not report any particular event that stopped them from eating the compote. Three volunteers left the study because of minor adverse digestive events, which it was thought might possibly be attributed to the compote.

### Study of gender difference

For all these previous criteria, no difference was observed between males and females

## Discussion

The main limit of this study is that it is not a double-blind randomized control trial versus a control compote; this would have made it possible to show that the addition of protein and fiber did not affect the taste of the compote and did not alter the subjects’ adherence compared with a standard compote. The use of such a model was not required from the standpoint of the expected results. The objective was whether this formulation could intrinsically secure adherence of elderly people and whether that adherence could be sustained over the medium term of 3 weeks in the real-life conditions of the subjects. This result appears to be demonstrate this, with hedonic scores varying between 4.6 and 4.1 out of a maximum of 6 and with 79.2 % of subjects who still found the compote ‘pleasant’ to ‘very pleasant’ after eating it every other day for 3 weeks. The quantities ingested by subjects are also meaningful and vary from 61.5 to 79.5 %, corresponding to 77–99 g, respectively, and an intake of 3.9–5.1 g of proteins and 4.1–5.3 g of fibers per day. It can also be observed that regular ingestion of a compote enhanced in protein and fibers does not produce digestive discomfort and that the rare undesirable effects were comparatively weakly ascribed to the compote. The impact on the fatigue score is negligible, showing the absence of any harmful effects on the volunteers. Moreover, in the latter connection a supplement of enhanced compote for 3 weeks is probably in any case not enough to produce a beneficial effect, particularly as the intake of proteins and fibers was not accompanied by a physical stimulation program and above all because the persons enrolled were not in a state of excessive fatigue, which made the scope for improvement comparatively modest. Identifying any effect of protein and fiber supplementation on fatigue would have required an entirely different protocol, whereas this study concentrated on the medium-term adherence of elderly people. The study may also be criticised for bearing on a small sample only and not providing guarantees as to the representative character of all of the people living in nursing homes. However, the group size was sufficient for observing any possible rejection.

The high level of adherence observed can in all likelihood be explained by a variety of factors. The first is probably the actual taste of apple and biscuit; this is reminiscent of apple pie, which is a flavor largely enjoyed by much of the population. It is of particular interest among elderly persons as the taste for sweet foods remains more marked for them than that for savory and bitter foods [13]. As the perception of tangy flavors declines among people fed in homes [13], the intake of the slightly tart apple flavor may contribute to that interest. In any event, and even if there is no means of making comparisons, the degree of adherence suggests that the addition of proteins and fiber does not reduce the subjects’ adherence. The study also shows that taking the compote at teatime is a viable alternative to lunchtime; this suggests the hypothesis that it could be eaten for breakfast.

In nutritional terms, the digestibility of NUTRALYS^®^ pea protein is 97.3 % with a PDCCAS (Protein Digestibility-Corrected Amino Acid Score) of 93 for adults [14]. Its branched amino acid (isoleucine, leucine and valine) content is important for muscle cells, while its digestibility profile ‘intermediate-fast protein’ is particularly suitable for elderly persons affected by sarcopenia. The rapid, but gradual increase in essential amino acids would presumably activate the synthesis of muscle proteins [15]. In addition, the inclusion of NUTRALYS^®^W hydrolyzed wheat gluten, a protein characterized as ‘fast’, can balance the amino acid profile of the compote by providing methionine and cysteine [16]. In this connection, the complementarity of wheat and pea proteins is ideal for this type of recipe. Similarly, NUTRIOSE^®^ soluble fiber has proven its beneficial effects with regard to modulating the colonic environment [17, 18] as related to colonic fermentation. This kind of fiber intake is important because, as the INCA 2 study showed, a mean fiber consumption of 19 g per day has been observed among senior citizens aged 55–79 years; this is below the EFSA recommendation of 25 g per day needed in particular to ensure an intestinal transit of 2–3 days and a normal stool frequency of once per day [19].

NUTRALYS^®^ pea protein, NUTRALYS^®^W hydrolyzed wheat gluten and NUTRIOSE^®^ soluble fiber may therefore be thought of as possessing all-round nutritional advantages for the elderly.

This study thus suggests that providing easily incorporable proteins in technological terms in various types of food matrices might allow useful diversification of protein and fiber intake by the elderly. Daily adherence by the volunteers might have been obtained if a different enriched fruit compote had been available to alternate with the apple compote. The same proteins might also be incorporated in foods other than desserts, thus multiplying the possible sources of protein supplements for the elderly. Work in this direction has already been undertaken on a brioche [20]. This is an important area of research because such diversification of protein supplementation is probably the most important way to contribute to maintaining muscle mass among the elderly and therefore their mobility. Far from liquid protein nutritional supplements, biscuits and other forms, these should be regarded as complements to each other, offering the wide range of foods needed to stimulate the appetites of the elderly.

## Conclusion

Nine out of ten volunteers appreciated the taste of fiber- and protein-enhanced compote; this taste adherence was maintained by eight out of ten volunteers over 3 weeks of regular consumption without change in hedonic satisfaction. Moreover, such consumption did not induce any adverse change in the digestive quality of life and did not produce any directly attributable adverse effects. Associated with traditional foodstuff, this innovative formulation provides an alternative solution to the usual nutritional supplements by diversifying food, thereby helping to elicit greater adherence among the elderly.

## References

[1] Pereira AF, Silva AJ, Matos Costa A (2013). Muscle tissue changes with aging. Acta Med Port.

[2] Montero-Fernández N, Serra-Rexach JA (2013). Role of exercise on sarcopenia in the elderly. Eur J Phys Rehabil Med.

[3] Morley JE (2012). Undernutrition in older adults. Fam Pract.

[4] AFSSA (Agence Française de Sécurité Sanitaire des Aliments) (2007) Apport en protéines: consommation, qualité, besoins et recommandations [Protein intake: dietary intake, quality, requirements and recommendations], p 461

[5] Fielding RA, Vellas B, Evans WJ (2011). Sarcopenia: an undiagnosed condition in older adults. Current consensus definition: prevalence, etiology, and consequences. International working group on sarcopenia. J Am Med Dir Assoc.

[6] Clark BC, Manini TM (2010). Functional consequences of sarcopenia and dynapenia in the elderly. Curr Opin Clin Nutr Metab Care.

[7] Paddon-Jones D (2009). Rasmussen BB Dietary protein recommendations and the prevention of sarcopenia. Curr Opin Clin Nutr Metab Care.

[8] Paddon-Jones D, Leidy H (2014). Dietary protein and muscle in older persons. Curr Opin Clin Nutr Metab Care.

[9] Lawless HT, Heymann H (1998). Sensory evaluation of food: principles and practices.

[10] Eypasch E, Williams JI, Wood-Dauphinee S (1995). Gastrointestinal quality of life index: development, validation and application of a new instrument. Troidl H Br J Surg.

[11] J. Gardenas et coll, Echelles et outils d’évaluation en médecine générale. Le Généraliste. Supplément du N°2187; Mars 2002

[12] Aguilova L, Sauzéon H, Balland É (2014). AGGIR scale: a contribution to specifying the needs of disabled elders. Rev Neurol (Paris).

[13] Toffanello ED, Inelmen EM, Imoscopi A (2013). Taste loss in hospitalized multimorbid elderly subjects. Clin Interv Aging.

[14] Yang H, Guerin-Deremaux L, Zhou L (2012). Evaluation of nutritional quality of a novel pea protein. Agro Food Ind Hi-Tech.

[15] Overduin J, Guérin-Deremaux L, Wils D, Lambers T (2015). NUTRALYS^®^pea protein: characterization of in vitro gastric digestion and in vivo gastrointestinal peptide responses relevant to satiety. Food Nutr Res.

[16] Dangin M, Boirie Y, Guillet C (2002). Influence of the protein digestion rate on protein turnover in young and elderly subjects. J Nutr.

[17] Lefranc-Millot C, Guerin-Deremaux L, Wils D (2012). Impact of a resistant dextrin on intestinal ecology: how altering the digestive ecosystem with NUTRIOSE^®^, a soluble fiber with prebiotic properties, may be beneficial for health. J Int Med Res.

[18] Hobden MR, Martin-Morales A, Guerin-Deremaux L (2013). In vitro fermentation of NUTRIOSE^®^ FB06, a wheat dextrin soluble fibre, in a continuous culture human colonic model system. PLoS One.

[19] EFSA Panel on Dietetic Products, Nutrition, and Allergies (NDA) (2010). Scientific opinion on dietary reference values for carbohydrates and dietary fibre. EFSA J.

[20] Allaert FA (2014). Comparaison de l’impact de la consommation du pain brioché G-Nutrition^®^ enrichi en protéines et vitamines et d’un complément nutritionnel oral liquide à forte densité protéique sur le statut nutritionnel des personnes âgées. Nutrition clinique et Métabolisme.

